# The microdrip method rapidly and efficiently enumerates bacterial colony-forming units in bovine milk

**DOI:** 10.3168/jdsc.2025-0788

**Published:** 2025-07-30

**Authors:** Kristi L. Jones, Nicole Adams, Anna M. Lundgren, Alexis Irvin, Ricardo C. Chebel, Aria Eshraghi

**Affiliations:** 1 Department of Large Animal Clinical Sciences, University of Florida, Gainesville, FL 32608; 2 Department of Infectious Diseases & Immunology, University of Florida, Gainesville, FL 32608; 3 Emerging Pathogens Institute, University of Florida, Gainesville, FL 32608; 4 Department of Oral Biology, University of Florida, Gainesville, FL 32608

## Abstract

In vivo microbial challenge models are an important tool to study the mechanistic details of bovine mastitis. A critical element of these studies is the enumeration of the bacterial load in milk to determine if interventions induce physiological changes that affect bacterial clearance. Herein, we use *Escherichia coli* P4 enumeration from milk in the context of mastitis microbial challenge models to show that use of a microdrip method reduces cost and time without negatively affecting rigor and reproducibility. We used *E. coli* P4–spiked milk samples as a model to test an alternative to *Escherichia* and costly standard plating methods. Importantly, linear regression analysis comparing the microdrip and standard plate count shows no difference between the methods or technicians, and Bland–Altman analysis shows enumeration via microdrip method has a slight positive bias, proportional across the tested concentrations compared with the standard method. Additionally, the microdrip method has a sensitivity of 10^3^ cfu/mL compared with 10^4^ cfu/mL for quantitative PCR. Economic analysis of consumable cost shows the microdrip method is nearly half as expensive compared with the standard plate method. Furthermore, these data also show milk samples can be stored at 4°C or −80°C without affecting colony-forming unit count, enabling batched sample processing. Taken together, the data presented here indicate that the microdilution and microdrip method can be used to decrease time and cost of *E. coli* P4 enumeration from milk without negatively affecting the rigor and reproducibility of the data. Future studies can develop this method for use in broader applications, such as validation of additional challenge pathogens for research purposes, clinical monitoring of mastitis cases, or bulk tank monitoring of pathogens.

## Highlights

• The microdrip method reduces time and cost of cfu enumeration in mastitis studies.
• This method has increased reproducibility and lower variability than the standard.
• The microdrip method sensitivity is similar to that of quantitative polymerase chain reaction.
• Milk samples can be stored for up to 7 days at 4°C or −80°C without changes in cfu.

## Graphical Abstract

**Figure:**
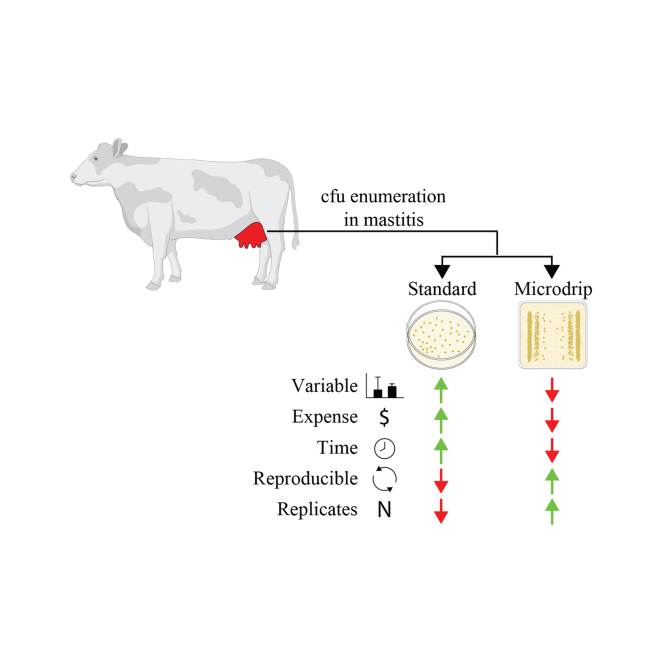
**Summary:** Investigating bacterially induced experimental mastitis requires reliable, precise, and reproducible enumeration of viable bacteria in milk. Standard microbiological methods to enumerate colony-forming units (cfu) are time consuming and expensive. In this work, we describe a microdrip method of cfu enumeration that not only reduces cost and time, but is also less variable, has increased reproducibility, and accommodates plating a greater number of technical replicates compared with standard bacterial enumeration methods. This suggests that the microdrip method is superior, considering statistical and practical considerations.

Bovine mastitis is an inflammatory response in the mammary glands, commonly caused by bacterial infections. Both clinical and subclinical infections are frequent and widespread. Prevalence of clinical mastitis on the cow level is estimated at 15% globally, 20% in the United States, and upward of 60% in some developing countries, resulting in substantial financial losses (Krishnamoorthy et al., 2021; USDA-APHIS, 2016; Belay et al., 2022). An estimated global annual cost of US$22 billion is associated with bovine mastitis, and North America alone has an estimated loss of US$3.6 billion (Rasmussen et al., 2024). These losses stem from decreased milk production, reduced milk quality, and high cost of treatment. Infusion of bacteria into the mammary gland is a robust and reproducible technique to study various aspects of mastitis, including host response to pathogen exposure, efficacy of immunization, and the impact of feed additives on mammary health (Moyes et al., 2014; Herry et al., 2017; Vailati-Riboni et al., 2021). *Escherichia coli* is a common gram-negative bacterial agent of environmental clinical mastitis (Morales-Ubaldo et al., 2023). Therefore, it is used to induce experimental mastitis for studies investigating physiological responses to gram-negative mammary infection (Blum et al., 2017; Lippolis et al., 2022). Although it is not the only pathogen used for intramammary challenge studies, *E. coli* is an ideal model for gram-negative experimental infection because it is cleared without pharmaceutical intervention within 72 h after exposure, thus reducing the use of antibiotics in the experimental herd (Blum et al., 2017; Lippolis et al., 2022). *Escherichia coli* strain P4 (O32:H37) was originally isolated from a case of bovine mastitis, and intramammary infusion of this strain reproduced the disease to fulfill Koch's postulates (Bramley, 1976). Subsequently, a standard protocol for intramammary infection of cows with *E. coli* P4 was established, and this protocol has been used in an extensive number of studies as a suitable model for experimental mastitis (Blum et al., 2017; Lippolis et al., 2022).

Currently, we lack a rapid, repeatable, inexpensive method for enumerating bacterial concentrations in milk from dairy cows. We used enumeration of *E. coli* P4 from milk in the context of an intramammary challenge study as a model to provide an alternative to time-consuming and costly standard plating methods. As described, the microdrip method is applicable to future challenge studies with the similar parameters. However, with further development and validation, it has the potential to be applied to routine culture of bulk tank and individual cow and quarter milk samples, in addition routine management of udder health where precise bacterial concentration data may not be necessary. Techniques to enumerate viable bacteria have gone largely unchanged for decades. Generally, serial dilutions are plated onto agar, incubated, and then colonies are counted, with the assumption that one colony originated from a single bacterial cell. This standard spread plate method is typically based on plating 100 µL of serially diluted samples, then counting a reasonable (20–200) number of colonies. The initial sample concentration is calculated from the colony count and the dilution factor. Due to sample variability, best practices require 3 technical replicates of each dilution, using the average value to calculate the colony-forming units per milliliter in the original sample (Sanders, 2012; Eden, 2014). Early work addressed these limitations by introducing a spiral plating method that does not require dilutions and was subsequently validated for use in raw ewe milk (Gilchrist et al., 1973; García-Armesto et al., 2002). However, this method requires specialized and expensive equipment that must be thoroughly cleaned between each sample, leading to limited sample throughput. Non-culture-based methods, such as flow cytometry and quantitative PCR, have been used to both identify bacterial species and enumerate from pure and mixed cultures (Mancusi and Trevisani, 2014; Ou et al., 2017). However, these techniques have disadvantages, including specialty equipment and expertise requirements or the inability to easily differentiate between viable and nonviable cells. Therefore, due to the relative ease, serial dilutions and plate counts remain the standard for bacterial enumeration. As early as the late 1960s, researchers investigated methods to minimize the dilution and plating volumes for studies to enumerate bacteria in milk (Fung and Lagrange, 1969). Subsequent studies have shown these lower-volume methods are generally easier to count due to the reduced total number of colonies to count, as 10 is considered a countable number of colonies with sufficient statistical power (Herigstad et al., 2001; Naghili et al., 2013). Additionally, multiple samples, dilutions, and replicates can be plated on a single plate, reducing time and consumables and thus decreasing cost.

Thebjecttive of our study was to determine if using the microdrip method is an effective and efficient method for enumeration of *E. coli* P4 from bovine milk. To this end, we evaluated (1) variability within and between biological replicates for standard and microdrip plating methods, (2) variability between technicians for both plating methods, (3) efficiency based on plating and counting time for both plating methods, (4) sensitivity compared with quantitative PCR (**qPCR**), and (5) economic analysis of plating methods based on cost of laboratory consumables.

To prepare bacteria for spiking into milk, *E. coli* P4 (provided by John Lippolis, USDA, Ames, IA) was streaked out on Luria–Bertani (**LB**) agar (Research Products International), and the following day, an isolated colony was selected and grown to mid-logarithmic phase in LB broth at 37°C while shaking. Bacteria were washed and resuspended in PBS, and the concentration was estimated by measuring the optical density at 600 nm. Commercially purchased homogenized and pasteurized milk was spiked with the resuspended bacteria to achieve ∼10^6^, 10^5^, 10^4^, and 10^3^ cfu/mL (n = 5 technical replicates per concentration). Spiked milk samples were split and half was given to one technician for enumeration via the standard spread plate method and the remainder given to a second technician for the microdrip method.

To mimic an experimental mastitis setting, where the technicians are unaware of the initial bacterial concentration in each sample, the technicians in this study were not given this information. Based on this lack of a priori knowledge, both technicians were required to serially dilute the samples and plate them to result in a countable number of colonies. In the standard method, each spiked milk sample was serially diluted 10-fold in microcentrifuge tubes, and 100 mL of the diluted samples was spread onto a circular Petri dish using sterilized glass beads (Figure 1A). For the microdrip method, a 12-channel micropipette was used to add 180 mL of LB into rows B through G of a sterile 96-well plate, and then 200 mL of the spiked milk samples was added to rows A and H (Figure 1B). The same multichannel pipette was used to serially dilute samples in row A into rows B through D by adding 20 mL of the source well into the subsequent well and mixing thoroughly by pipetting. Subsequently, an 8-channel pipette was used to draw up 10 mL of the samples in each column and drip onto a tilted square Petri dish. All plates were incubated at 37°C, and the next day, plates were screened for a countable number of colonies. For each sample, the technicians counted a single circular Petri dish or a row of the microdrip plate. Each technician completed 3 rounds of enumeration using each method (n = 6 total biological replicates).

**Figure 1:**
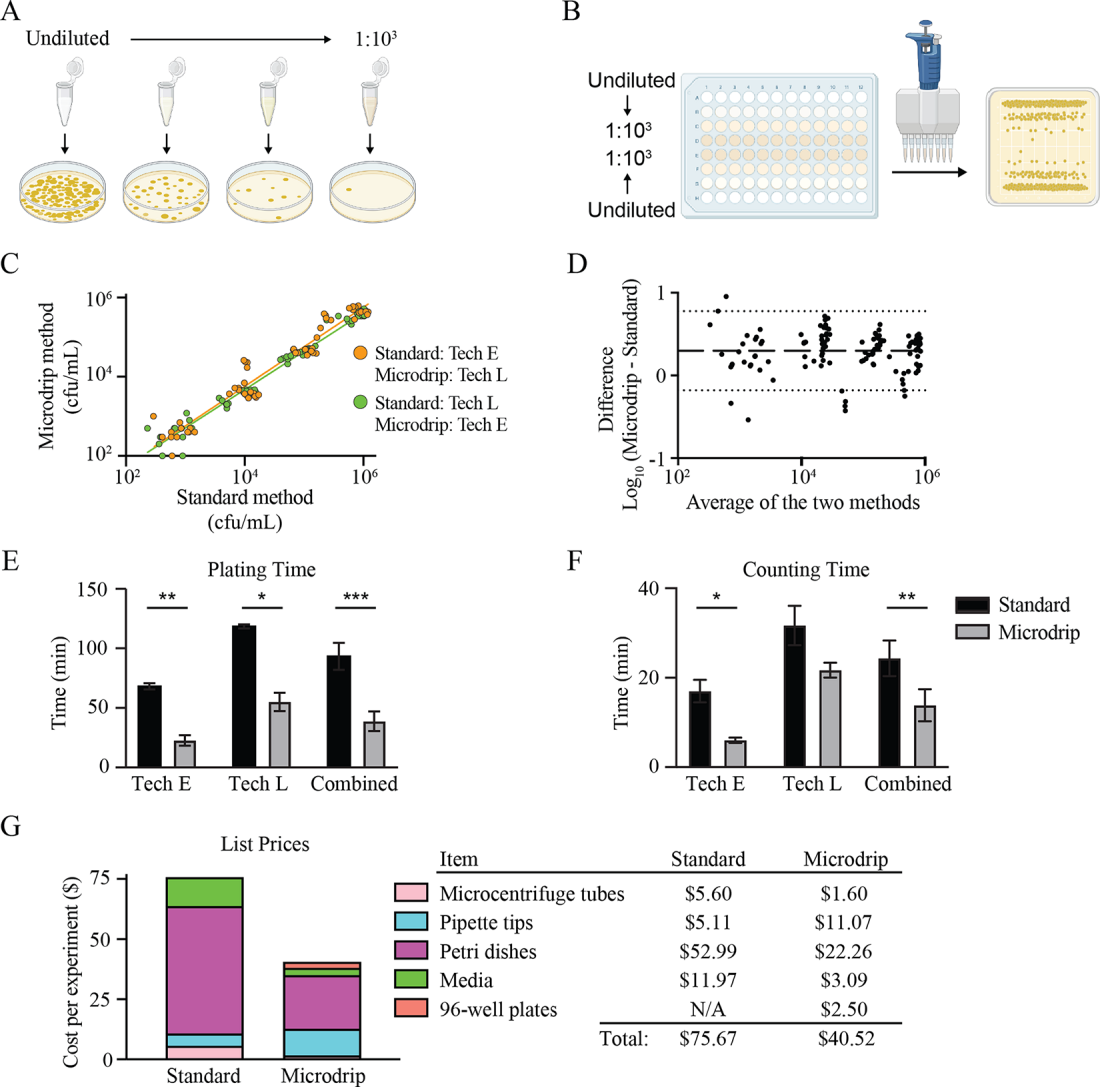
Comparison of the bacterial colony-forming unit enumeration methods. (A) In the standard method, each milk sample is diluted in a series of microcentrifuge tubes, and 100 mL of multiple dilutions for each sample is spread onto Petri dishes with a spreader, followed by incubation and enumeration of colony-forming units by counting colonies. (B) In the microdrip method, up to 24 milk samples are diluted in a 96-well plate with a micropipette, and 10 mL of each diluted sample is dropped onto a square Petri dish and allowed to run down the surface of the plate, followed by incubation and colony-forming unit enumeration by counting colonies on one lane for each sample. (C) Colony-forming unit enumeration performed with the 2 methods by 2 different technicians (Tech). In the first 3 biological replicates, technician E performed the standard method and technician L the microdrip method (orange dots and line, R^2^ = 0.93). In the next 3 biological replicates, the roles were reversed (green dots and line, R^2^ = 0.97). This dataset represents a total of 6 biological replicates with 5 technical replicates per condition. (D) Bland–Altman analysis of log-transformed colony-forming unit enumeration data from the 2 methods. The horizontal dashed line above zero indicates bias, and dotted lines indicate 95% limits of agreement. Comparison of the duration of time required for (E) plating and (F) counting in the 2 methods, presented for each of the technicians separately and combined. Data represent a total of 6 biological replicates, and bars represent means and SEM (paired *t*-test: *P < 0.05, **P < 0.01, ***P < 0.001). (G) Current list prices for the consumable supplies and reagents required for the standard and microdrip colony-forming unit enumeration methods. N/A: not applicable.

We compared the lower limit of quantification (**LOQ**) of the microdrip method to qPCR. First, we optimized a SYBR Green (Thermo Fisher Scientific)-based qPCR protocol for *E. coli* enumeration based on amplification efficiency. We tested 3 previously established primer sets that target *E. coli* P4 genomic DNA (ybbW: 5'-TGATTGGCAAAATCTGGCCG-3' and 5'-GAAATCGCCCAAATCGCCAT-3'; uidA1: 5'-CGGAAGCAACGCGTAAACTC-3' and 5'-TGAGCGTCGCAGAACATTACA-3'; and uidA2: 5'-CGGTGATATCGTCCACCCAG-3' and 5'-TGGATCGCGAAAACTGTGGA-3'; Ohad et al., 2015; Walker et al., 2017; Lamba et al., 2020). Using the optimized primer set we assessed LOQ, defined as the lowest spiked *E. coli* concentration (cfu/mL milk) to produce a statistically different cycle threshold than a sample of DNA extracted from non-spiked milk.

To determine the impact of storage temperature on *E. coli* P4 in bovine milk, we spiked milk to ∼4 × 10^3^ cfu/mL. Then we aliquoted these samples and stored them at 4 different temperatures: room temperature (∼22°C), 4°C, −20°C, and −80°C. At 1, 3, 5, 7, and 14 d after incubation, aliquots were pulled from storage at each temperature, and colony-forming unit enumeration was performed using the microdrip method to evaluate changes in bacterial burden in the milk. Twelve technical replicates were counted for each incubation temperature, and 3 biologically independent replicates were performed. Changes in the concentration of viable bacteria were recorded and compared across storage temperatures to identify conditions that best preserved the initial bacterial concentration.

Linear regression analysis of the resulting data shows no difference between technicians (R^2^ = 0.93) or methods (R^2^ = 0.97), indicating that data generated using the microdrip method are similar to those generated by standard colony-forming unit enumeration (Figure 1C). Comparison of 2 methods by Bland–Altman analysis revealed a slight bias that is proportional across the tested concentrations, indicating that the microdrip method enumerates samples slightly higher than the standard method and that this bias is consistent across concentrations (Bland and Altman, 1986; Figure 1D). However, it is unclear which of the 2 methods is most accurate from this analysis. Additionally, the variability between the methods increased at the lowest tested concentration (10^3^ cfu/mL). Based on these data, we concluded that the microdrip method can accurately enumerate colony-forming units and is interchangeable with the standard method.

Analysis of repeatability within a biological replicate performed by one technician revealed that variability within technical replicates (% CV) is greatest for both methods at low concentrations (Table 1). We observed a notable increase in variability when using the microdrip method to enumerate a sample spiked with 10^3^ cfu/mL, which we attributed to this method's smaller pipetting volumes. When reproducibility across independent biological replicates was compared, the standard method displayed much higher variability than the microdrip method, for which the variability was below 10% in all tested concentrations (Table 1). Both methods displayed increased variability across replicates as the expected concentrations decreased. These data indicate that the microdrip method is more consistent than the standard method across biological and technical replicates.

**Table 1:** Analysis of variability within and between biological replicates

Spike concentration (cfu/mL)	Variability within biological replicates % CV		Reproducibility between biological replicates % CV	
	Standard	Microdrip	Standard	Microdrip
10^6^	1.06	1.42	5.92	1.82
10^5^	0.58	1.5	12.42	2.47
10^4^	2.28	1.31	28.31	4.68
10^3^	3.63	11.06	38.04	8.56

Because the data yielded from the microdrip method are interchangeable with and less variable than standard spread plating, we sought to determine if the microdrip method is more efficient and economical. Both technicians displayed a significant decrease in the amount of time necessary to plate 24 samples when using the microdrip method (*P* < 0.01, Figure 1E). The time required for dilution and inoculation in the microdrip method is less than half of the standard method, effectively increasing the throughput of this phase of the protocol by 2-fold. In addition to plating time, we measured the time necessary to count the colonies produced by each method (Figure 1F). Colony counting time was reduced with the microdrip method by almost half compared with the standard method (*P* < 0.01). This difference was mainly explained by the fact that technician E required substantially less time to count colonies in the microdrip method compared with the standard, whereas the time required to count colonies by technician L was not affected by method. Further studies involving a broader pool of technicians may be performed to confirm the generalizability of the observed time savings and efficiency gains with the microdrip system. In total, these results suggest that the microdrip method is an accurate and more rapid approach to enumerating colony-forming units than the standard method.

These data indicate that the accuracy and efficiency of the microdrip method is superior; however, the small volume of the inoculum calls into question whether the LOQ of this method is sufficient for use in mastitis studies. Thus, we compared the LOQ of the microdrip method to qPCR, a highly sensitive method to quantify bacterial DNA. We compared 3 primer sets to find the most sensitive for comparison to the microdrip method. Amplification efficiency of all 3 primer sets was greater than 92%, and the uidA2 primer set had the greatest difference in cycle threshold values between samples containing 600 pg and no DNA, indicating this primer set is the most sensitive of the 3 (data not shown). Based on these results, we determined the LOQ of this primer set by amplifying DNA extracted from milk spiked with *E. coli* P4. The optimized qPCR reproducibly distinguished between milk containing 10^4^ cfu/mL and a sample containing no bacteria, suggesting that this is the LOQ. To produce accurate and precise colony-forming unit enumeration data, the minimal countable number of colonies in the microdrip method is 10 cfu in a 10 µL volume; therefore, the LOQ of the microdrip method is 10^3^ cfu/mL (Figure 1C). It is important to note that we determined the LOQ and not the limit of detection (**LOD**) because experimental challenge studies require colony-forming units per milliliter values and not binary presence or absence data. Of note, not surprisingly, the LOQ determined here is greater than the LOD threshold set by the National Mastitis Council (2020) for diagnosis of mastitis; therefore, we propose use of the micodrip method for challenge samples collected at early time points (time 6–60 h after infusion) when the count is expected to be greater than 10^3^ cfu/mL (Lippolis et al., 2022). Further development of this method is possible to improve the LOD and potentially expand applicability. Comparison of the LOQ of qPCR and the microdrip method demonstrates that they have similar sensitivity, with the microdrip method being slightly more sensitive. A notable drawback of qPCR is that it cannot discern between DNA extracted from viable and nonviable bacteria. This presents a considerable limitation in qPCR of samples that come from animals that have robust immune systems that actively function to clear bacterial infections. Therefore, we conclude the microdrip method is a more robust bacterial quantification assay compared with qPCR for this type of sample.

Although reproducibility and sensitivity of an assay to enumerate colony-forming units is of utmost importance to maintain rigorous standards, it is also important to consider the economics of the methods. To compare the cost of the microdrip method to the standard method, we quantified the materials used to enumerate 24 samples and calculated the costs associated with each method (Figure 1G). The majority of the cost for both methods is consumable plasticware attributed mostly to Petri dishes and pipette tips. The total cost of bacterial enumeration using the standard method was $75.67, and the cost to enumerate the same number of samples using microdrip method was 46% less at $40.52. Furthermore, the mass of expended consumable plasticware in the microdrip method is only 30% of what is used in the standard method, suggesting a noteworthy environmental benefit (data not shown). Taken together, these data demonstrate that the microdrip method is considerably more cost-effective than the standard method. Taking into account our analysis showing the microdrip method produces data consistent with the standard method in less time and reduced cost the implementation of this approach will benefit dairy microbiology studies.

Another challenge that bovine mastitis researchers face is the need to enumerate colony-forming units in many samples harvested in a short period of time, so in addition to our time comparison between the methods, we investigated the impact of storage temperature and time on enumeration. To do this, it is essential to elucidate conditions that do not affect bacterial concentrations in milk. At just 1 d after incubation, storage at room temperature resulted in increased bacterial load, whereas storage of samples at −20°C resulted in a substantial loss of colony-forming units; thus, these conditions are not feasible options for preservation of viable bacteria (Figure 2). In contrast, storage at 4°C and −80°C maintained the initial bacterial concentrations throughout the duration tested, indicating these temperatures are the suitable for sample storage. We hypothesize that the natural milk fats may provide a cryoprotectant effect at −80°C, but this property is ineffective at −20°C due to a slower freezing rate, resulting in formation of ice crystals that attenuate bacterial viability.

**Figure 2:**
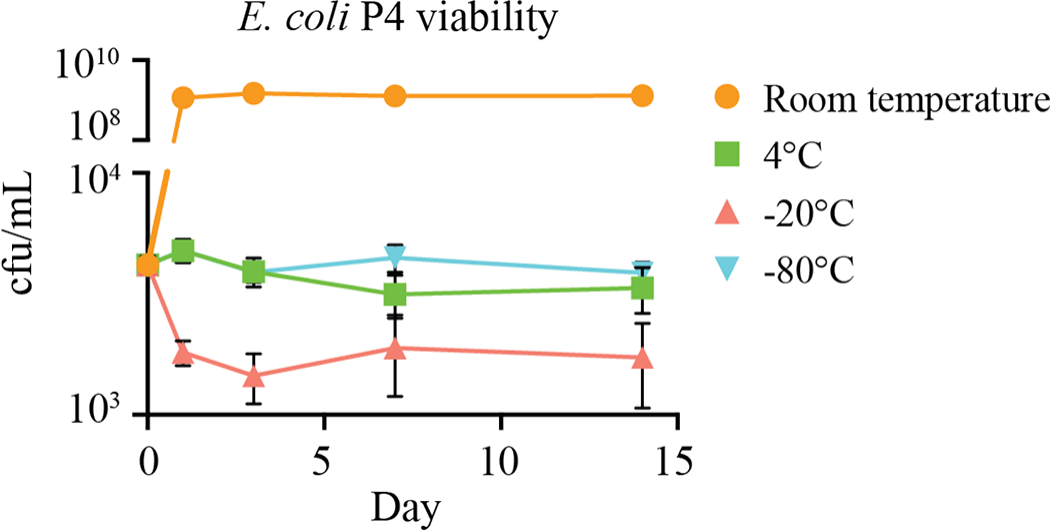
The microdrip method enumerates bacteria in bovine milk stored at 4°C or −80°C for an extended period of time. Enumeration of *Escherichia coli* P4 spiked into bovine milk stored at different temperatures by using the microdrip method reveals that samples stored at 4°C or −80°C do not change significantly, whereas storage at room temperature allows growth and viability is lost at −20°C. These data represent 12 technical replicates per condition and 3 biological replicates. Points and error bars represent means and SEM.

The data reported here are a comprehensive evaluation of the microdrip method to enumerate *E. coli* P4 from milk considering analysis of variability, sensitivity, financial cost, and technician time. Consistent with previous reports, our data indicate consistency between the standard plating method and a plating of smaller volumes. These data demonstrate within and between run variability is generally lower using the microdrip method compared with the standard method. Additionally, our data shows there is minimal variability between technicians. Furthermore, our analysis indicated the values (cfu/mL) calculated from both methods are comparable, and there is a consistent overestimate of the microdrip method compared with the standard method; this can be further examined to develop correction factors. We also report a comparison with qPCR, a non-culture-based method of enumeration. Based on the results of these studies, we recommend use of the microdrip method for milk samples ≥10^3^ cfu/mL, which is consistent with samples collected at most time points in *E. coli* P4 mastitis challenge experiments. To further mitigate time restraints and streamline sample handling during these experiments, our data indicate that storage of bacteria at 4°C or −80°C does not alter colony-forming unit enumeration; thus, samples can be collected and stored for subsequent colony-forming unit enumeration in batches.

We evaluated variability within and between biological replicates, as well as between technicians, and report that the microdrip method yields reliable and reproducible quantitative data equivalent to the standard spread plate method. As expected, our data indicate the microdrip method has key advantages, including dramatic reductions in plating and counting time and a notable decrease in the cost of required consumables. Additionally, we found that storage of milk samples at 4°C or −80°C did not alter bacterial counts (cfu/mL), so samples collected over an extended period can be banked for later analysis. We conclude that the reliable and reproducible data and the increased efficiency and economic savings make the microdrip method preferable to the standard method for enumerating *E. coli* P4 in milk in most circumstances.
